# Nutrition and Exercise Interventions to Improve Body Composition for Persons with Overweight or Obesity Near Retirement Age: A Systematic Review and Network Meta-Analysis of Randomized Controlled Trials

**DOI:** 10.1016/j.advnut.2023.04.001

**Published:** 2023-04-06

**Authors:** Doris Eglseer, Mariella Traxler, Stefan Embacher, Lea Reiter, Josje D. Schoufour, Peter J.M. Weijs, Trudy Voortman, Yves Boirie, Alfonso Cruz-Jentoft, Silvia Bauer, the SO-NUTS consortium

**Affiliations:** 1 Medical University of Graz, Institute of Nursing Science, Graz, Austria; 2 Medical University of Graz, Institute for Medical Informatics, Statistics and Documentation, Graz, Austria; 3 Faculty of Sports and Nutrition, Centre of Expertise Urban Vitality, Amsterdam University of Applied Sciences, Amsterdam, the Netherlands; 4 Department of Nutrition and Dietetics, Amsterdam University Medical Centers, Amsterdam Public Health Institute, VU University, Amsterdam, the Netherlands; 5 Division of Human Nutrition and Health, Wageningen University & Research, Wageningen, the Netherlands; 6 Department of Epidemiology, Erasmus MC University Medical Center Rotterdam, Rotterdam, the Netherlands; 7 University Clermont Auvergne, Human Nutrition Unit, INRA, CRNH Auvergne, CHU Clermont-Ferrand, Clinical Nutrition Department, Clermont-Ferrand, France; 8 Servicio de Geriatría, Hospital Universitario Ramón y Cajal (IRYCIS), Madrid, Spain

**Keywords:** obesity, overweight, retirement, network meta-analysis, caloric restriction, resistance training, body composition, body mass index, fasting

## Abstract

The retirement phase is an opportunity to integrate healthy (nutrition/exercise) habits into daily life. We conducted this systematic review to assess which nutrition and exercise interventions most effectively improve body composition (fat/muscle mass), body mass index (BMI), and waist circumference (WC) in persons with obesity/overweight near retirement age (ages 55–70 y). We conducted a systematic review and network meta-analysis (NMA) of randomized controlled trials, searching 4 databases from their inception up to July 12, 2022. The NMA was based on a random effects model, pooled mean differences, standardized mean differences, their 95% confidence intervals, and correlations with multi-arm studies. Subgroup and sensitivity analyses were also conducted. Ninety-two studies were included, 66 of which with 4957 participants could be used for the NMA. Identified interventions were clustered into 12 groups: no intervention, energy restriction (i.e., 500–1000 kcal), energy restriction plus high-protein intake (1.1–1.7 g/kg/body weight), intermittent fasting, mixed exercise (aerobic and resistance), resistance training, aerobic training, high protein plus resistance training, energy restriction plus high protein plus exercise, energy restriction plus resistance training, energy restriction plus aerobic training, and energy restriction plus mixed exercise. Intervention durations ranged from 8 wk to 6 mo. Body fat was reduced with energy restriction plus any exercise or plus high-protein intake. Energy restriction alone was less effective and tended to decrease muscle mass. Muscle mass was only significantly increased with mixed exercise. All other interventions including exercise effectively preserved muscle mass. A BMI and/or WC decrease was achieved with all interventions except aerobic training/resistance training alone or resistance training plus high protein. Overall, the most effective strategy for nearly all outcomes was combining energy restriction with resistance training or mixed exercise and high protein. Health care professionals involved in the management of persons with obesity need to be aware that an energy-restricted diet alone may contribute to sarcopenic obesity in persons near retirement age.

This network meta-analysis is registered at https://www.crd.york.ac.uk/prospero/ as CRD42021276465.

**Statement of significance**

This comprehensive network meta-analysis uniquely focuses on people near retirement age which have a great potential for implementing healthy nutrition and exercise habits during this transition phase. The methodology allowed an indirect comparison to be included in the statistical model, therefore further enhancing the significance and coverage of the model.

## Introduction

Overweight and obesity are serious disorders with prevalence rates among older Europeans of about 60% and 20%, respectively [1]. In the United States, the obesity prevalence is even higher at around 40% [2]. These rates have steadily increased worldwide over the last 40 y in men and women [[2], [3], [4], [5]]. Its high prevalence and serious social, economic, and health consequences make it one of the major global health problems [[6], [7], [8]]. Obesity is a major risk factor for several diseases, including type 2 diabetes mellitus, coronary artery disease, cerebral vascular disease, arterial hypertension, dyslipidemia, and several types of cancer. All of these conditions contribute to a reduction in both the quality of life and life expectancy [7,8]. For example, an increase in a society’s BMI by 2 points shortens the life expectancy by 0.7 to 1 y [9]. Furthermore, obesity is accompanied by burdens such as falls, disability, or care dependency, especially in older adults [7,8,10].

Obesity is characterized by excessive fat accumulation [11] that often occurs during the process of aging. This especially occurs in persons aged 45 to 70 y, with a weight peak observed at middle age, i.e., 50 to 65 y of age [8,[12], [13], [14]]. Aging is accompanied not only by a gradual increase in body fat (BF) stores but also a decrease in muscle mass, muscle function, and water retention. Simultaneously suffering from obesity and the progression of the aging process can lead to sarcopenic obesity, a condition that combines the loss of muscle mass, strength, and function with an increase in adiposity [15,16]. This affects a remarkably large group of people, with prevalence rates of up to 33.5% observed, e.g., in the older US population [17], which suggests that many obese people simulateously suffer from sarcopenia. Because loss of muscle mass is often accompanied by an increase in fat mass, body weight may remain stable [18, 19], meaning that a stable or even decreasing body weight can mask increasing adiposity [19].

The retirement age is generally between 48 and 67 y in the Organization for Economic Co-operation and Development countries [20]; this is exactly in the age range during which the previously described major changes in body composition occur. Therefore, the retirement phase represents a window of opportunity to decelerate the associated deterioration in body composition. This phase is a period of change. In most cases, this change is not gradual, but occurs abruptly one day when a person no longer needs to go to work and needs to start redesigning their everyday life [12]. A recent longitudinal study showed that 61% of people in this age group changed their lifestyle during the retirement phase [21]. People that changed their lifestyle by reducing risk factors for obesity, such as poor diet or inactivity, showed smaller physical declines over time in later life. This finding underlines the great potential for implementing healthy nutrition and exercise habits and thus increasing the disability-free life expectancy in the retirement phase [21].

Nutrition and exercise interventions are considered as first-line therapies for treating individuals with overweight and obesity [4,11,22]. These should not only be effective in reduction of BF but also in preserving muscle mass. This is even more important in older adults to prevent the occurence of disability [11]. Several systematic reviews have summarized different nutrition and exercise interventions in older persons with overweight and obesity [[23], [24], [25], [26], [27], [28], [29], [30]], but we identified only one that focused on people near retirement age [23]. This review, however, examined the effectiveness of dietary interventions on healthy eating habits and not on obesity parameters such as body composition or anthropometric parameters.

Thus, prior to the current review, a comprehensive systematic review and network meta-analysis (NMA) of the effects of nutrition and exercise interventions in persons near retirement age was lacking. Such an analysis is highly beneficial for the scientific community and clinical practice, because the results can provide recommendations for effective interventions for people who are overweight or obese in this target group. The specific aim of conducting this systematic review using NMA methodology was to assess which nutrition and exercise interventions are most effective for improving the body composition (fat mass and muscle mass), BMI, and waist circumference (WC) in persons with overweight or obesity near retirement age (55 to 70 y of age).

## Methods

This systematic review and NMA of randomized controlled trials (RCTs) has been registered in PROSPERO (International Prospective Register of Systematic Reviews, https://www.crd.york.ac.uk/prospero/, identifier CRD42021276465). The principles of the Preferred Reporting Items for Systematic Review and Meta-analysis 2021 were applied for reporting NMAs [31].

### Search strategy

We conducted a comprehensive literature search to identify RCTs that had been published in PubMed (MEDLINE), EMBASE via OVID, CINAHL via EBSCO Host, and the Cochrane Central Register of Controlled Trails (CENTRAL) via the Cochrane Library from the inception of these databases and up to July 12, 2022. No language or calendar date restrictions were set. In addition, we conducted a manual search of reference lists from eligible studies and Google Scholar. We also searched for gray literature in the online platforms available via https://clinicaltrials.gov/ and the WHO International Clinical Trials Registry (ICTRP). The literature review was conducted by 2 authors (MT, SB) independently.

We used the following search terms: “obesity,” “diet,” “exercise,” “train∗,” and “physical activity.” Additionally, we used the following MeSH terms: “obesity,” “diet,” and “exercise.” As part of the search strategy, the search terms were combined using the Boolean operators AND and OR. We applied filters for RCTs and the respective age group (middle-aged and aged) and made small adaptations for each database searched (see Supplementary Table 1).

### Study selection

**FIGURE 1:**
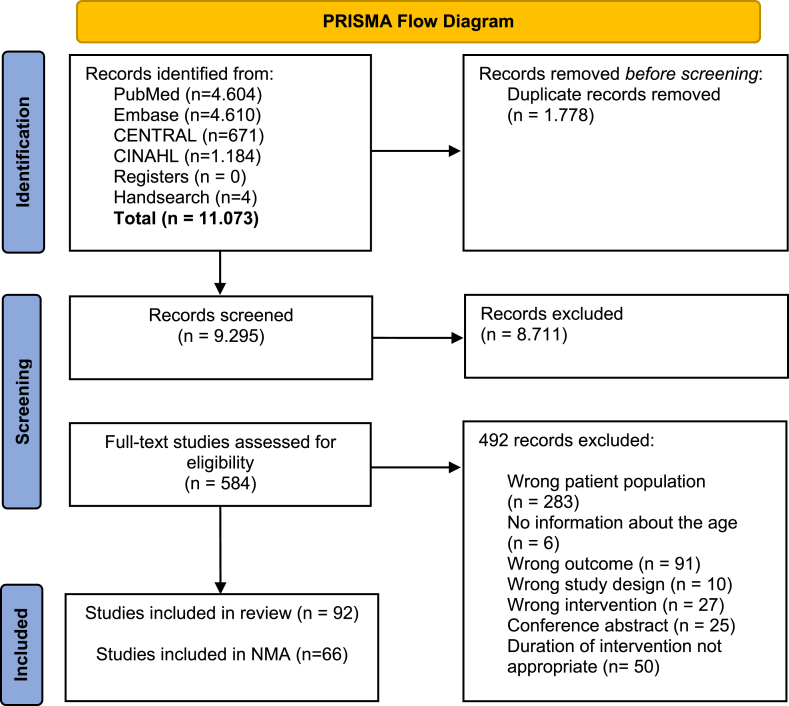
PRISMA flow diagram of the literature review and study selection process

The study selection process was conducted with the systematic review software COVIDENCE (Veritas Health Innovation). We included RCTs using a parallel or crossover design, based on our predefined Population, Intervention, Control, Outcome (PICO) question (Table 1). We excluded studies focusing on persons with specific health conditions, such as cancer, type 2 diabetes, heart failure, or pulmonary diseases, as well as studies with specific target groups, such as soccer players or truck drivers. We further excluded RCTs with pharmaceutical or behavioral interventions other than nutrition or activity interventions for obesity and studies that lacked a clear description of the intervention and weight maintenance studies. Title and abstract screening as well as full-text screening were performed based on inclusion and exclusion criteria by 2 authors independently of one another (MT, SB, DE). The numbers and reasons for the exclusion of studies are listed in the flow chart (see Figure 1). Any disagreements were resolved by a discussion involving a third person (DE).

**TABLE 1:** Inclusion criteria for RCTs based on the PICO question

Parameters	Search strategy
Participants	Community dwelling, persons with overweight or obesity (BMI > 25 to 40 kg/m^2^) near the retirement age (mean age in the single studies between 55 and 70 y, independent of the CI)
Intervention	Any nutrition or exercise intervention for a duration between 8 wk and 6 mo
Control	No intervention, any other intervention
Outcomes	BF in kg, %BF, LBM/FFM, BMI, WC
Study design	Randomized controlled trials

Abreviations: BF, body fat; WC, waist circumference.

### Data extraction and quality assessment

Two reviewers independently extracted data from the final included full-text articles, and disagreements were resolved by a discussion involving a third person. We generated a standardized data extraction template, including the study characteristics, patient characteristics, intervention(s), adherence to the intervention(s), and patient outcomes. Prior to the data extraction, we piloted the template of 2 studies to identify and edit possible shortcomings in the template. The methodological quality of the RCTs was assessed by 2 independent reviewers using the Cochrane Risk of Bias tool, referring to the Cochrane Handbook for Systematic Reviews of Interventions [32].

### Data synthesis: interventions

Based on the different interventions used in the identified studies, we created 12 pragmatic intervention/control categories: *1*) no intervention, *2*) energy restriction (i.e., caloric restriction of 500 to 1000 kcal), *3*) energy restriction plus high protein intake (1.1–1.7 g/kg body weight/d), *4*) intermittent fasting (5:2 diet), *5*) mixed exercise (aerobic and resistance training), *6*) resistance training, *7*) aerobic training, *8*) high-protein intake plus resistance training, *9*) energy restriction plus high-protein intake plus exercise, *10*) energy restriction plus resistance training, *11*) energy restriction plus aerobic training, and *12*) energy restriction plus mixed exercises (aerobic and resistance training combined). Studies that could not be assigned to any of these categories and studies that included study arms comparing similar interventions that would have been in the same category were described narratively.

### Statistical analysis

The change in the outcome measures was used for the statistical analysis. This change was calculated as the mean difference (MD) between the baseline and follow-up for each treatment group. Most included studies provided means and standard deviations at baseline and for specific follow-up dates. We then calculated the MD and the standard deviation of change referring to the Cochrane Handbook for Systematic Reviews of Interventions and assuming a correlation of 0.85 [33]. This value was chosen because we observed a high correlation between the baseline and follow-up measures in studies where baseline, follow-up, and change measures were reported. If not directly provided, further steps were taken to calculate the corresponding values. This included the use of *P* values and confidence intervals; where ranges were reported, we applied the same methodology as Hozo et al. [34]. The NMAwas based on a random effects model, and correlations in multi-arm studies were considered [35]. The common heterogeneity variance $\tau^{2}$ used in the random effects model was estimated with a generalized DerSimonian-Laird estimator [36]. To assess inconsistency, the between-designs *Q*-value was calculated based on a full design-by-treatment interaction model for random effects [37]. If studies had more than one arm belonging to the same combined treatment group, we combined the corresponding means, standard deviations, and sample sizes [38]. We used Egger’s test to appraise the data for potential publication bias, i.e., to identify asymmetry in the funnel plot [39]. For models where we compared effects within the same outcome measure, we used the MD. In models where we compared more than one outcome measure, we used the standardized mean difference (SMD) to assure comparability. For one model, we prioritized data, choosing the first available outcome measures in the following order: BF in %, BF in kg, muscle mass (LBM/ FFM), WC, then BMI. Here, we considered the different direction of positive effects for the fat mass and muscle mass.

Additionally, we performed several subgroup and sensitivity analyses. First, we analyzed data stratified by sex, from studies with only females, only males, or reporting on both sexes separately. Second, we distinguished between the duration of the intervention(s) being less or equal to 14 wk or being more than 14 wk. Third, we repeated analyses excluding all studies identified as having a high risk of bias. Fourth, we grouped the interventions even further into the following 3 categories: nutrition, exercise, or nutrition plus exercise. In all subgroup analyses, we used one fat mass as an outcome measure (body fat or BF in %) and muscle mass as an outcome measure (LBM/FFM), deciding on a case-by-case basis and using the ones that were reported more frequently in order to include the highest number of studies in the analysis. A 2-sided *P* value of less than 0.05 was considered to be statistically significant. All analyses were performed in R (Version 4.1.3).

## Results

We identified and screened 11,073 records, from which 92 RCTs met the selection criteria (Figure 1). Of these, 6 RCTs included interventions that could not be assigned to one of the 12 intervention/control categories (i.e., different types of oil supplementation, vegan diet, and Mediterranean diet), 2 RCTs included less than 8 participants, and 18 RCTs compared interventions within the same intervention category (e.g., comparison of energy restriction with target 500 kcal and 1000 kcal). For these reasons, we included 66 studies [[40], [41], [42], [43], [44], [45], [46], [47], [48], [49], [50], [51], [52], [53], [54], [55], [56], [57], [58], [59], [60], [61], [62], [63], [64], [65], [66], [67], [68], [69], [70], [71], [72], [73], [74], [75], [76], [77], [78], [79], [80], [81], [82], [83], [84], [85], [86], [87], [88], [89], [90], [91], [92], [93], [94], [95], [96], [97], [98], [99], [100], [101], [102], [103], [104], [105]] that involved nutrition and exercise interventions in persons with overweight/obesity near retirement age and included a total of 4957 participants in the final NMA. Figure 1 illustrates the literature review process.

### Characteristics of the included RCTs

Of all 92 identified studies, we identified 21 RCTs that focused on nutrition interventions alone, 25 RCTs that focused on exercise interventions alone, and 46 RCTs that combined nutrition and exercise interventions. Overall, we identified 82 different interventions described in the studies, which we assigned to 12 categories created based on the interventions used in the primary studies.

Most studies included both male and female participants (*n* = 51), followed by studies that only included female participants (*n* = 36). The included studies were conducted in 19 different countries, and mostly in the United States (*n* = 49), see Table 2. The sample sizes ranged from 11 to 543 participants. The intervention period ranged from 8 wk to 6 mo, with the most common intervention periods being 12 wk (*n* = 33 or 36%), 26 wk (*n* = 21 or 23%), and 16 wk (*n* = 11 or 12%). Most of the exercise studies (*n* = 19 or 76%) performed training sessions 3 times a week for 20 to 60 min. The most common nutrition intervention was energy restriction (with or without meal replacement, mostly with a reduction in fat content and/or carbohydrates, including low- and very low-calorie diets).

**TABLE 2:** Study characteristics of included studies and their assigned intervention groups

Author (y)	Country	Sample Size	Sex	No. of Arms	Duration of Intervention	BC measurement	Intervention [Intervention Group]	Adherence	Incl. in NMA
Combined Nutrition and Exercise Interventions									
Amamou et al. [41] 2017	Canada	26	Male + Female	2	16 wk	DXA	1. Energy restriction + dietary high protein [3] 2. Energy restriction + high protein + RT (3x/wk) [9]	Not mentioned	Yes
Amati et al. [42] 2008	United States	64	Male + Female	3	16 wk	DXA	1. Energy restriction (500-1000 kcal) [2] 2. Aerobic training (> 3x/wk, walking, cycling, etc.) [7] 3. Energy restriction + AT (3x/wk) [11]	Not mentioned	Yes
Avila et al. [43] 2010	United States	27	Male + Female	2	10 wk	ADP	1. DASH Diet education [2] 2. DASH Diet education + RT (3x/wk)[10]	Intervention 1 dietary interv.: 85% Intervention 2 dietary interv: 98%, RT: 96%	Yes
Beebe et al. [44] 2013	United States	26	Female	2	16 wk	No BC measured	1. DASH diet education [2] 2. DASH diet education + Tai chi (3x/wk) [10]	Not mentioned	Yes
Bopp et al. [45] 2008	United States	70	Female	3	20 wk	DXA	1. Energy restriction (300-400 kcal) [2] 2. Energy restriction + high-intensity AT (3x/wk) [11] 3. Energy restriction + low-intensity AT (3x/wk) [11]	Intervention 1 Not mentioned Intervention 2 Dietary compliance: 100.1% +/- 0.4 Intervention 3 Not mentioned	Yes
Bouchard et al.[46] 2009	United States	46	Female	4	12 wk	DXA	1. Resistance training (3x/wk) [6] 2. Energy restriction (500-1000 kcal) [2] 3. Energy restriction + RT (3x/wk) [10] 4. No Intervention (education, normal diet) [1]	Not mentioned	Yes
Brennan et al. [107] 2020	United States	61	Male + Female	3	26 wk	DXA	1. No Intervention (education, normal diet) 2. Energy restriction (500-1000 kcal) 3. Energy restriction + exercise (4-5x/wk AT+ RT)	Not mentioned	No
Brochu et al. [47] 2009	Canada	107	Female	2	16 wk	DXA	1. Energy restriction (500-1000 kcal) [2] 2. Energy restriction + RT (3x/wk) [10]	Intervention 1 80-90% Intervention 2 80-90%	Yes
Deibert et al. [48] 2011	Germany	35	Male	3	12 wk	Skinfold measurement	1. No intervention (education, normal diet) [1] 2. Resistance training (2x/wk) [6] 3. RT (2x/wk) + high protein (supplement) [8]	Intervention 1 90% Intervention 2 90% Intervention 3 90%	Yes
Dubé et al. [49] 2011	United States	16	Male + Female	2	16 wk	DXA	1. Energy restriction (500-1000 kcal) [2] 2. Aerobic training (> 3x/wk, walking, cycling, etc.) [7]	Not mentioned	Yes
Evans et al. [50] 2021	United States	61	Female	3	26 wk	DXA	1. Energy restriction + high protein (supplement) [3] 2. Energy restriction + high protein + exercise (3x/wk AT + RT) [9] 3. Energy restriction + Exercise (3x/wk AT + RT) [12]	Intervention 1 dietary adherence not mentioned Intervention 2 75% exercise Intervention 3 75% exercise	Yes
Felix-Soriano et al. [108] 2021	Spain	85	Female	4	16 wk	DXA	1. Olive oil (30 ml or capsule form) 2. Omega-3 fatty acids 3. Omega-3 fatty acids RT (2x/wk) 4. Olive oil + RT (2x/wk)	Intervention 1 95% Intervention 2 95% Intervention 3 95% Intervention 4 95%	No
Galbreath et al. [51] 2018	United States	54	Female	3	14 wk	BIA, DXA	1. Exercise (3x/wk AT +RT) [5] 2. Energy restriction + high carbs + exercise (3x/wk AT+RT) [12] 3. Energy restriction + high protein + exercise (3x/wk AT + RT) [9]	Intervention 1 70% Intervention 2 70% Intervention 3 70%	Yes
Grossman et al. [109] 2018	United States	11	Female	2	16 wk	DXA	1. Energy restriction + HIIT (3x/wk) 2. Energy restriction + AT (3x/wk)	Intervention 1 100% Intervention 2 60%	No
Hays et al. [110] 2004	United States	34	Male + Female	3	14 wk	BOD POD	1. No intervention (education, normal diet) 2. Low-fat, complex-carbohydrate (HI-CHO) 3. Low fat, high comlex carbs + exercise (4x/wk HI-CHO + EX)	Not mentioned	No
Haywood et al. [53] 2018	Australia	117	Male + Female	3	12 wk	DXA	1. Aerobic training (> 3x/wk, walking, cycling, etc.) [9] 2. VLCD + AT (3x/wk) [5] 3. Energy restriction + AT (3x/wk) [5]	Not mentioned	Yes
Hsu et al. [106] 2021	Taiwan	69	Male + Female	3	12 wk	DXA	1. No intervention (education, normal diet) [1] 2. High-intensity training (HIIT) [7] 3. Energy restriction + high protein + HIIT (3x/wk) [9]	No Intervention Not mentioned Intervention 2 90% Intervention 3 94%	Yes
Jefferson et al. [52] 2015	United States	32	Male + Female	2	20 wk	DXA	1. Resistance training (3x/wk) [7] 2. Energy restriction + RT (3x/wk) [10]	Intervention 1 84% Intervention 2 86%	Yes
Jo et al. [111] 2019	United States	11	Male + Female	2	14 wk	DXA	1. VLCD (very low-calorie diet) + Optifast + high protein 2. VLCD + RT (3x/wk) + Optifast	Not mentioned	No
Kelly et al. [112] 2014	United States	24	Male + Female	2	12 wk	DXA, CT	1. Low-glycemic index diet (LoGIX) + exercise (5x/wk) 2. High-glycemic index diet (HiGIX) + exercise (5x/wk)	Intervention1 diet + exercise: 97% Intervention2 diet + exercise: 97%	No
McNeil et al. [54] 2015	Canada	93	Female	2	26 wk	DXA	1. Energy restriction (500-1000 kcal) [2] 2. Energy restriction + RT (3x/wk) [10]	Not mentioned	Yes
Messier et al. [55] 2010	Canada	107	Female	2	26 wk	DXA	1. Energy restriction (500-1000 kcal) [2] 2. Energy restriction + RT (3x/wk) [10]	Not mentioned	Yes
Mulya et al. [113] 2017	United States	20	Male + Female	2	12 wk	DXA	1. High-glycemic index diet + exercise (HiGIX) 2. Low-glycemic index diet (LoGIX) + exercise (5x/wk)	Intervention 1 83.3% Intervention 2 84.3%	No
Muollo et al. [114] 2019	Italy	38	Male + Female	2	24 wk	DXA	1. Supervised Nordic walking (3x/wk) + energy restriction 2. Supervised traditional walking (> 3x/wk) + energy restriction	Not mentioned	No
Muollo et al. [115] 2021	Italy	27	Male + Female	2	24 wk	DXA	1. Unsupervised Nordic walking (3x/wk) + energy restriction 2. Unsupervised traditional walking (> 3x/wk) + energy restriction	Intervention 1 81.4% Intervention 2 80.2%	No
Nicklas et al. [58] 2019	United States	155	Male + Female	3	20 wk	DXA	1. Aerobic training (> 3x/wk, walking, cycling, etc.) [7] 2. Moderate energy restriction + AT (-250 kcal) [11] 3. Energy restriction + AT (3x/wk) [11]	Intervention 1 85.8% Intervention 2 Exercise: 89.9%; 99.2% Intervention 3 Exercise 91.2%; 100%	Yes
Nicklas et al. [57] 2015	United States	126	Male + Female	2	20 wk	DXA	1. Resistance training (3x/wk) [6] 2. Energy restriction + RT (3x/wk) [10]	Intervention 1 attendance: 86% Intervention2 attendance: 89%	Yes
Nicklas et al. [56] 2009	United States	112	Female	3	20 wk	DXA, CT	1. Energy restriction (500-1000 kcal) [2] 2. Energy restriction + AT with moderate intensity (3x/wk) [11] 3. Energy restriction + AT (3x/wk) at vigorous intensity [11]	Intervention 1 dietary compliance: 99.8% (SD 1.4%) Intervention2 dietary compl.: 100.3% (SD 1.8); exercise: 92.6% (SD 5.5) Intervention 3 dietary compl.: 100.4% (SD 1,7); exercise: 90% (SD 8.7)	Yes
Sakurai et al. [59] 2013	Japan	66	Male + Female	4	12 wk	BC analyzer	1. Hot bathing + education + exercise (RT + band + aerobic, 2x/wk) [5] 2. education + exercise (RT + band + aerobic 2x/wk) [5] 3. hot bathing 20 min [1] 4No intervention (education, normal diet) [1]	Not mentioned	Yes
Santanasto et al. [60] 2011	United States	36	Male + Female	2	26 wk	DXA, CT	1. Energy restriction + exercise (3x/wk AT + RT) [12] 2. Exercise (3x/wk AT + RT) [5]	Not mentioned	Yes
Shah et al. [61] 2009	United States	18	Male + Female	2	26 wk	DXA	1. Energy restriction (500-1000 kcal) [2] 2. Energy restriction + exercise (3x/wk AT + RT) [12]	Not mentioned	Yes
Solomon et al. [116] 2013	United States	20	Male + Female	2	12 wk	DXA	1. Aerobic training (> 3x/wk, walking, cycling, etc.) + low-glycemic diet 2. Aerobic training (> 3x/wk, walking, cycling, etc.) + high-glycemic diet	Not mentioned	No
St-Onge et al. [74] 2013	Canada	89	Male + Female	2	26 wk	DXA	1. Energy restriction (500-1000 kcal) [2] 2. Energy restriction + RT (3x/wk) [10]	Not mentioned	Yes
Valente et al. [73] 2011	United States	27	Male + Female	2	10 wk	ADP	1. DASH diet education [2] 2. RT (3x/wk) + DASH diet education [10]	Intervention 1 85% Intervention 2 98%	Yes
van Gemert et al. [117] 2015	Netherlands	243	Male + Female	3	16 wk	DXA	1. No intervention (education, normal diet) 2. Energy restriction (500-1000 kcal) 3. Exercise (4 h/wk AT + RT)	Intervention 1 80% Intervention 2 80% Intervention 3 80%	No
Verreijen et al. [71] 2017	Netherlands	100	Male + Female	4	10 wk	ADP (BOD POD)	1. Energy restriction (500-1000 kcal) [2] 2. Energy restriction + dietary high protein [3] 3. Energy restriction + high protein + RT (3x/wk) [9] 4. Energy restriction + resistance training (3x/wk) [10]	Intervention 1 Not mentioned Intervention 2 mean adherence to exercise program: 2.9 +- 0.3 times/wk Intervention 3 mean adherence to exercise program: 2.9 +- 0.3 times/wk Intervention 4 Not mentioned	Yes
Verreijen et al. [72] 2015	Netherlands	60	Male + Female	2	13 wk	DXA	1. Energy restriction + high protein + RT (3x/wk) [3] 2. Energy restriction + RT (3x/wk) [10]	Intervention 1 food consupmtion: 91%; exercise program: 72% Intervention 2 food consumption: 97%; exercise program: 88%	Yes
Villareal et al. [70] 2017	United States	160	Male + Female	4	26 wk	DXA	1. No intervention (education, normal diet) [1] 2. Energy restriction + AT (3x/wk) [11] 3. Energy restriction + RT (3x/wk) [10] 4. Energy restriction + exercise (3x/wk AT+RT) [12]	Not mentioned	Yes
Wang et al. [69] 2015	United States	70	Female	2	20 wk	DXA	1. Energy restriction + AT (3x/wk) [11] 2. Energy restriction (500-1000 kcal) [2]	Not mentioned	Yes
Wasserfurth et al. [67] 2020	Germany	134	Male + Female	4	12 wk	BIA	1. No intervention (education, normal diet) [1] 2. Exercise (AT +RT 2x/wk) [5] 3. Exercise + German Nutrition Society [5] 4. Exercise + normal diet + 2 g/d Calanus finmarchichus oil (Shellfish) [5]	Not mentioned	Yes
Waters et al. [67] 2021	United States	160	Male + Female	4	26 wk	DXA	1. No intervention (education, normal diet) [1] 2. Energy restriction + AT (3x/wk) [11] 3. Energy restriction + RT (3x/wk) [10] 4. Energy restriction + exercise (AT + RT 3x/wk) [12]	Not mentioned	Yes
Weiss et al. [66] 2021	United States	52	Male + Female	3	12 wk	DXA	1. Energy restriction (500-1000 kcal) [2] 2. Exercise (AT + RT frequency not mentioned) [5] 3. Energy restriction + exercise (AT + RT frequency not mentioned) [11]	Not mentioned	Yes
Yassine et al. [65] 2009	United States	24	Male + Female	2	12 wk	Hydrostatic weighing, CT	1. Aerobic training (> 3x/wk, walking, cycling, etc.) [7] 2. Energy restriction + AT (3x/wk) [11]	Intervention 1 94% Intervention 2 94%	Yes
Yoshimura et al. [64] 2014	Japan	75	Male + Female	2	12 wk	Underwater weighing	1. Energy restriction (500-1000 kcal) [2] 2. Energy restriction + AT (3x/wk) [11]	Intervention 1 Diet only: 97% Intervention 2 Exercise: 81%, diet: 93%	Yes
You et al. [63] 2004	United States	34	Female	2	26 wk	DXA, CT	1. Energy restriction (500-1000 kcal) [2] 2. Energy restriction + AT (3x/wk) [11]	Intervention 1 diet class: 80% Intervention 2 diet + ex: 78%; exercise sessions: 78%	Yes
You et al. [62] 2006	United States	45	Female	3	20 wk	DXA	1. energy restriction (500-1000 kcal) [2] 2. Energy restriction + low-intensity AT (3x/wk) [11] 3. Energy restriction + high-intensity AT (3x/wk) [5]	Intervention 1 Not mentioned Intervention 2 exercise compl: 92.3 ± 1.7% Intervention 3 exercise compl: 87.9 ± 2.3%	Yes
Exercise Interventions									
Ballor et al. [75] 1996	United States	18	Male + Female	2	12 wk	Underwater weighing	1. Resistance training (3x/wk) [6] 2. Aerobic training (> 3x/wk, walking, cycling, etc.) [7]	Not mentioned	Yes
Bocalini et al. [76] 2012	Brazil	44	Female	4	12 wk	Skinfold measurement	1. No intervention (education, normal diet) [1] 2. Resistance training (3x/wk) [6] 3. No intervention (education, normal diet) [1] 4. Resistance training (3x/wk) [6]	Not mentioned	Yes
Boukabous et al. [120] 2019	Canada	18	Female	2	8 wk	DXA	1. High-intensity training (75 min/wk HIIT) 2. Moderate intensity training (150 min/wk MICT)	Intervention 1 92.7% Intervention 2 92.7%	No
Carneiro et al. [121] 2021	Brazil	40	Female	2	15 wk	DXA	1. Low-intensity RT (3x/wk) 2. High-intensity resistance training (3x/wk)	Not mentioned	No
Cavalcante et al. [77] 2018	Brazil	57	Female	3	12 wk	DXA	1. Resistance training (2x/wk) [6] 2. Resistance training (3x/wk) [6] 3. No intervention (education, normal diet) [1]	Intervention 1 session attending ≥ 85% Intervention 2 session attending ≥ 85% No intervention Not mentioned	Yes
Conley et al. [79] 2018	Australia	23	Male	2	26 wk	No BC measured	1. 5:2 diet [4] 2. Energy restriction (500-1000 kcal) [2]	Intervention 1 82% Intervention 2 83%	Yes
Faramarzi et al. [80] 2018	Iran	40	Female	4	8 wk	Skinfold measurement	1. Endurance training followed by strength training [5] 2. Strength training followed by endurance training [5] 3. Exercise (3x/wk AT + RT) [5] 4. No intervention (education, normal diet) [1]	Not mentioned	Yes
Fritz et al. [81] 2018	Spain	63	Female	3	8 wk	BIA, DXA	1. Elastic tubes with handles (ETG 2x/wk) [6] 2. Elastic band traditional (EB 2x/wk) [6] 3. No intervention (education, normal diet) [1]	Intervention 1 91.6 ± 3.3% Intervention 2 93.3 ± 3.1% No intervention Not mentioned	Yes
Izzicupo et al. [122] 2017	Italy	30	Female	2	12 wk	Skinfold	1. Traditional walking (> 3x/wk) 2. Nordic walking	Not mentioned	No
Kallings et al. [82] 2009	Sweden	101	Male + Female	2	26 wk	BIA	1. Exercise (frequency not mentioned AT + RT) [5] 2. No intervention (education, normal diet) [1]	Not mentioned	Yes
Kim et al. [83] 2019	Korea	20	Male	2	12 wk	BIA	1. No intervention (education, normal diet) [1] 2. Exercise (3x/wk AT + RT) [5]	Not mentioned	Yes
Li et al. [84] 2021	China	29	Male + Female	3	12 wk	DXA	1. No intervention (education, normal diet) [1] 2. High-intensity training (3x/wk HIIT) [7] 3. Vigorous-intensity continuous training (3x/wk VICT) [7]	Not mentioned	Yes
Nunes et al. [85] 2019	Brazil	24	Female	2	12 wk	DXA	1. Exercise (3x/wk AT + RT) [5] 2. HIIT (3x/wk) [6]	Not mentioned	Yes
Park et al. [86] 2015	Korea	20	Female	2	12 wk	BC analyzer	1. Exercise (3x/wk AT + RT) [5] 2. No intervention (education, normal diet) [1]	Not mentioned	Yes
Park et al. [87] 2020	Korea	20	Male	2	12 wk	BIA	1. No intervention (education, normal diet) [1] 2. Moderate exercise (3x/wk AT +RT) [5]	Not mentioned	Yes
Phillips et al. [88] 2012	United States	23	Female	2	12 wk	Skinfold measurement	1. Resistance training (3x/wk) [6] 2. No intervention (education, normal diet) [1]	Intervention 1 attendance exercise: 100% No intervention attendance: 90%	Yes
Puengsuwan et al. 2020 [89]	Thailand	55	Female	2	15 wk	Skinfold measurement	1. No intervention (education, normal diet) [1] 2. Resistance training (3x/wk) [6]	No intervention Not mentioned Intervention 2 exercise training: 90%	Yes
Ribeiro et al. [90] 2020	Brazil	33	Female	2	8 wk	DXA	1. No intervention (education, normal diet) [1] 2. Resistance training (3x/wk) [6]	No intervention Not mentioned Intervention 1 participating in sessions ≥ 85%	Yes
Rossi et al. [91] 2016	Brazil	70	Female	3	16 wk	DXA	1. No intervention (education, normal diet) [1] 2. Aerobic training (> 3x/wk, walking, cycling, etc.) [7] 3. Exercise (AT + RT 3x/wk) [5]	Not mentioned	Yes
Siu et al. [95] 2021	Hong Kong	543	Male + Female	3	12 wk	No BC measured	1. No intervention (education, normal diet) [1] 2. Exercise (3x/wk AT + RT) [5] 3. Tai chi [6]	No intervention Not mentioned Intervention 1 class attendance: 67% Intervention 2 class attendance: 70%	Yes
Sjögren et al. [92] 2012	Sweden	73	Male + Female	2	26 wk	BIA	1. No intervention (education, normal diet) [1] 2. Exercise (3x/wk AT + RT) [6]	Not mentioned	Yes
Stewart et al. [93] 2005	United States	104	Male + Female	4	26 wk	DXA	1. No intervention (education, normal diet) [1] 2. Exercise (3x/wk AT + RT) [5]	No intervention 90% Intervention 1 90%	Yes
Tomeleri et al. [94] 2016	Brazil	38	Female	2	8 wk	DXA	1. Resistance training (3x/wk) [6] 2. No intervention (education, normal diet) [1]	No intervention Not mentioned Intervention 1 session participating ≥ 85%	Yes
Church et al. [78] 2009	United States	411	Female	4	26 wk	Skinfold measurement	1. No intervention (education, normal diet) [1] 2. 4 kcal/kg/wk - 3 to 4 training sessions [7] 3. 8 kcal/kg/wk - 3 to 4 training sessions [7] 4. 12 kcal/kg/wk - 3 to 4 training sessions [7]	No intervention Not mentioned Intervention 1 exercise: 99.5% Intervention 2 exercise: 99.3 Intervention 3 exercise: 99.2%	Yes
Irwin et al. [104] 2003	United States	173	Female	2	12 wk	DXA	1. Aerobic training (> 3x/wk, walking, cycling, etc.) [7] 2. No intervention (education, normal diet) [1]	Not mentioned	Yes
Nutritional Interventions									
Backx et al. [103] 2016	Netherlands	61	Male + Female	2	12 wk	DXA	1. Energy restriction (500-1000 kcal) [2] 2. Energy restriction + dietary high protein [3]	Not mentioned	Yes
Barbour et al. [123] 2015	Australia	63	Male + Female	2	12 wk	DXA	1. High oleic peanut consumption (male: 84 g/female: 56 g/d) 2. No intervention (education, normal diet)	Intervention 1 80% No intervention Not mentioned	No
Barnard et al. [118] 2022	United States	62	Male + Female	2	16 wk	DXA	1. Mediterranean diet 2. Low-fat Vegan diet	Intervention 1 84% Intervention 2 84%	No
Barnard et al. [119] 2005	United States	59	Female	2	14 wk	BOD POD	1. Low-fat Vegan diet 2. No intervention (education, normal diet)	Not mentioned	No
Beaver et al. [102] 2015	United States	24	Male + Female	2	12 wk	DXA, CT	1. Energy restriction + normal soy protein + meal replacement [2] 2. Energy restriction (500-1000 kcal) [2]	Intervention 1 Self-reported compliance to dietary interv: 97.5 ± 3.3% Intervention 2 Self-reported compliance to dietary interv: 92.2 ± 9.3%	Yes
Beaver et al. [100] 2019	United States	96	Male + Female	2	26 wk	DXA	1. Energy restriction + high protein + meal replacement [3] 2. No intervention (education, normal diet) [1]	Intervention 1 attendance at educational sessions: 88%; self-reported meal replacement: 92.7% No intervention attendance at educational sessions: 84%	Yes
Dengo et al. [101] 2010	United States	36	Male + Female	2	12 wk	DXA, CT	1. No intervention (education, normal diet) [1] 2. Energy restriction (500-1000 kcal) [2]	Not mentioned	Yes
Dennis et al. [133] 2010	United States	48	Male + Female	2	12 wk	DXA	1. Energy restriction + 500 ml water prior to each daily meal 2. Energy restriction (500-1000 kcal)	Intervention 1 Water intake compliance: 90 ± 2%	No
Englert et al. [99] 2021	Germany	54	Female	2	12 wk	BIA	1. Energy restriction + normal protein + meal replacement [2] 2. Energy restriction + high protein + meal replacement [3]	Not mentioned	Yes
Goss et al. [125] 2020	United States	34	Male + Female	2	8 wk	DXA	1. Very low calorie and carbohydrate (< 10%) 2. Low-fat diet	Not mentioned	No
Ilich et al. [126] 2019	United States	135	Female	3	26 wk	DXA	1. No intervention (education, normal diet) 2. Energy restriction + calcium + vitamin D supplement 3. Energy restriction (500-1000 kcal) + low-fat dairy diet	No intervention compliance with suppl: 73% Intervention 2 compliance with suppl: 73% Intervention 3 compliance with placebo: 82.7%	No
Katz et al. [127] 2012	United States	46	Male + Female	2	8 wk	No BC measured	1. Walnuts 56 g 2. No intervention (education, normal diet)	Not mentioned	No
Kristensen et al. [128] 2012	Denmark	72	Female	2	12 wk	DXA	1. Energy restriction + refined wheat 2. Energy restriction + whole-grain wheat	Intervention 1 compl. with provided food: 91.5% Intervention 2 compl. with provided food: 94.2%	No
Njike et al. [129] 2017	United States	32	Male + Female	2	12 wk	BIA	1. Typical conventional snack food (200 kcal) 2. Nut-based snack bars (NBSB 200 kcal)	Not mentioned	No
Ogilvie et al. [98] 2021	United States	34	Female	2	26 wk	DXA	1. Energy restriction (500-1000 kcal) [2] 2. Energy restriction + dietary high protein [3]	Not mentioned	Yes
Porter Starr et al. [97] 2019	United States	39	Male + Female	2	26 wk	BOD POD	1. Energy restriction (500-1000 kcal) [2] 2. Energy restriction + dietary high protein [3]	Not mentioned	Yes
Serra et al. [96] 2019	United States	82	Male + Female	2	26 wk	DXA	1. Energy restriction + high protein + meal replacement [2] 2. No intervention (education, normal diet) [1]	Intervention 1 attendance to education: 88% self-reported compl: 93% No intervention attendance to education. session: 84%	Yes
Shapses et al. [130] 2004	United States	58	Female	4	25 wk	DXA	1. Energy restriction + calcium 1,000 mg/d 2. Energy restriction (500-1000 kcal) 3. Energy restriction + calcium 1,000 mg/d (Slim fast) 4. Energy restriction + Slim Fast	Intervention 1 90% Intervention 2 90% Intervention 3 85% Intervention 4 85%	No
Teng et al. [105] 2013	Malaysia	56	Male	2	12 wk	Body composition analyzer	1. No intervention (education, normal diet) [1] 2. Energy restriction (300-500) + 2x Muslim Sunnah Fasting/wk [4]	Not mentioned	Yes
Wien et al. [131] 2003	United States	65	Male + Female	2	24 wk	BIA	1. Formula-based LCD+ almond 84 g/d 2. Formula-based LCD + self-selected diet with complex carbs + safflower oil	Not mentioned	No
Christensen et al. [124] 2011	Denmark	192	Male + Female	2	16 wk	BIA	1. Very low-calorie diet (VLCD) 2. Low-energy diet (LED)	Intervention 1 after 8 wks: 91%/after 16 wks: 90% Intervention 2 8 wks: 94%; 16 wks: 93%	No

Abbreviations: ADP, air-displacement plethysmograph; AT, aerobic training; BC, body composition; LCD, low-calorie diet; RT, resistance training.

Intervention groups: [1] No intervention, [2] energy restriction (300-1000 kcal + meal replacement), [3] energy restriction + high protein, [4] 5:2 diet, [5] mixed exercise (AT+RT), [6] resistance training, [7] aerobic training, [8] resistance training + high protein, [9] energy restriction + high protein + exercise (mainly RT), [10] energy restriction + resistance training, [11] energy restriction + aerobic training, [12] energy restriction + mixed exercise.

### Risk of bias

**FIGURE 2:**
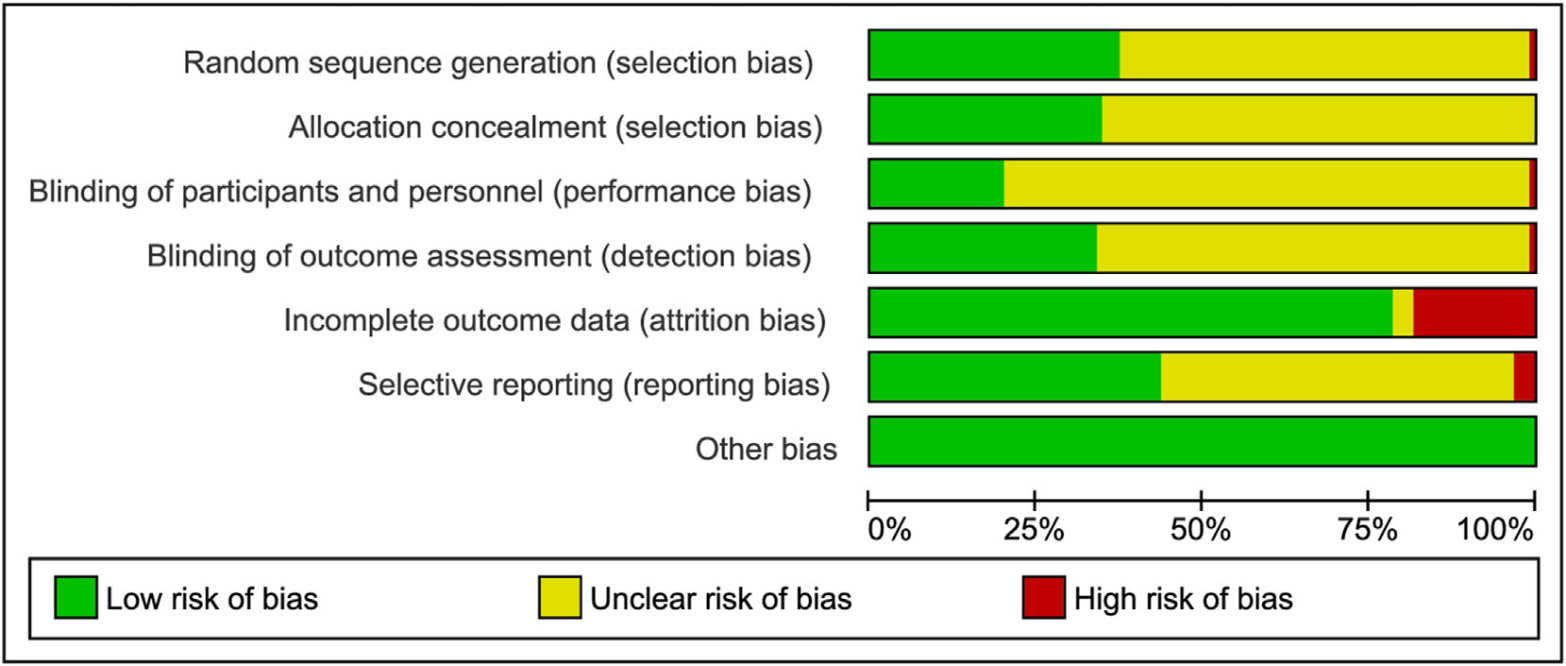
Risk of bias summary.

Overall, 19 RCTs (21 %) were rated as having a high risk of bias for at least one domain. Thirty-four studies (37%) were judged as having a low risk of bias in the domain random sequence generation, whereas 32 studies (35%) had a low risk of bias in the domain allocation concealment. The assessment of participant and personnel blinding revealed that 18 studies (20%) had a low risk of bias, and 31 studies (34%) had a low risk of bias for blinding the outcome assessor. Seventy-two (78%) of the studies had a low risk for presenting incomplete outcome data, and 40 (43%) had a low risk for selective reporting. The Egger’s test results for publication bias were not significant for all outcomes (*P* < 0.05). A summary of risk of bias assessment is provided in Figure 2.

### NMA

**FIGURE 3:**
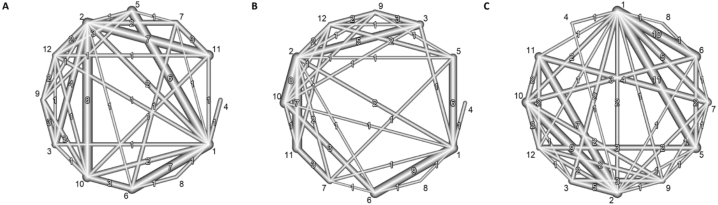
Network graphs comparing the structure of the network regarding (A) body fat in kg, (B) LBM/FFM, and (C) ALL according to the outcome prioritization. The numbers within the graphs represent the numbers of direct comparisons, while the thickness of the lines is proportional to the inverse standard error of the estimates. The numbers outside the graphs represent the intervention numbers as follows: 1) no intervention, 2) energy restriction, 3) energy restriction plus high-protein intake, 4) 5:2 diet, 5) mixed exercise (aerobic and resistance training), 6) resistance training, 7) aerobic training, 8) resistance training plus high-protein intake, 9) energy restriction plus high protein and exercise, 10) energy restriction plus resistance training, 11) energy restriction plus aerobic training, and 12) energy restriction plus mixed exercises.

**FIGURE 4:**
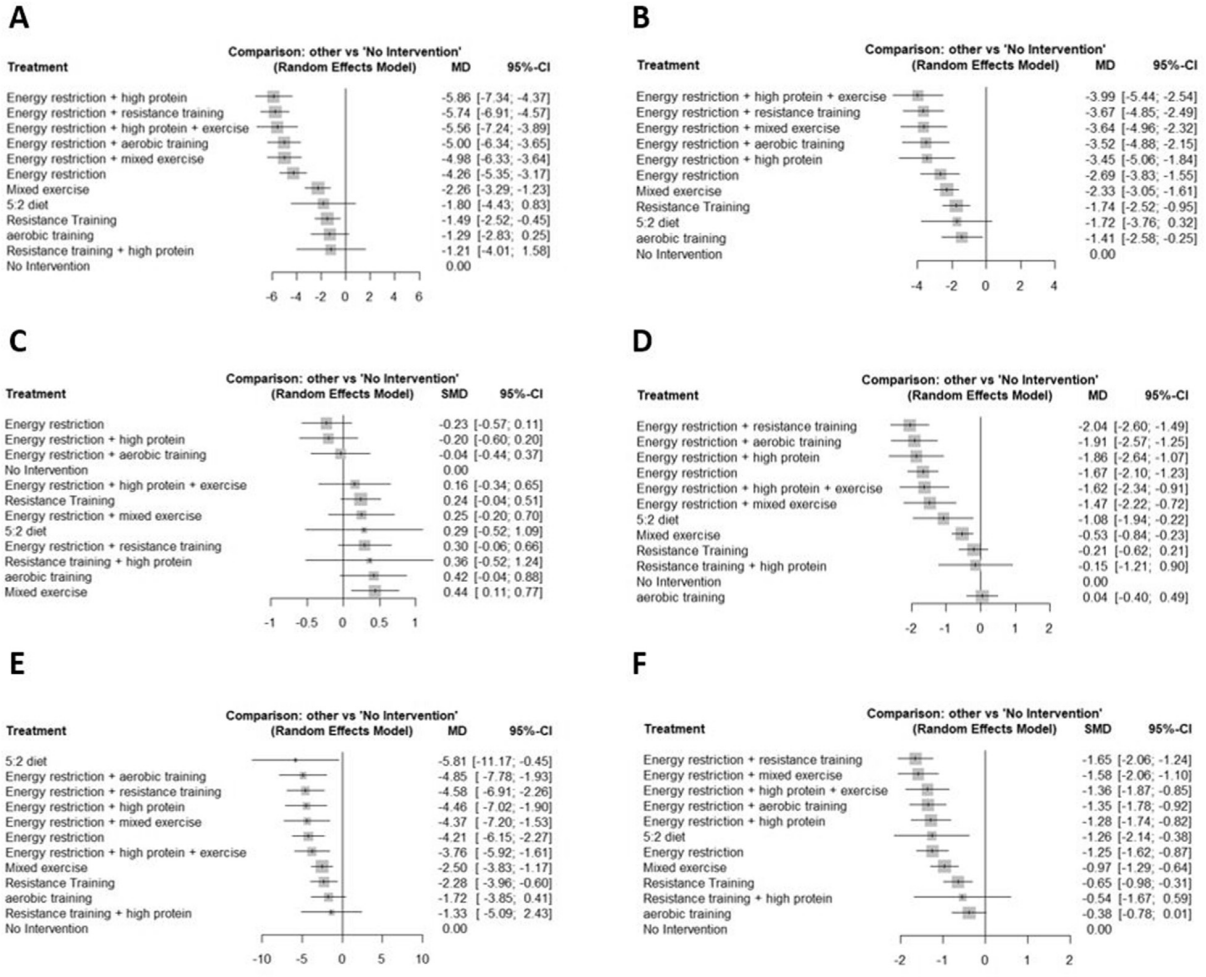
Summary effect estimates of the different nutrition and exercise interventions on (A) BF in kg, (B) %BF, (C) LBM/FFM, (D) BMI, (E) WC and (F) ALL according to the outcome prioritization. BF, body fat; WC, waist circumference.

We conducted the NMA model separately for the 5 outcomes (%BF, BF in kg, LBM/FFM, WC, BMI) and one model using all outcomes. The network geometry for the outcomes BF mass in kg and LBM/FFM, as well as the network geometry using all studies according to the predefined prioritization of outcomes is presented in Figure 3. All other network geometries can be found in Supplementary Figure 1. By grouping interventions into specific treatment groups, we obtained a dense network that enabled us to make many direct comparisons. The network where we used the prioritization shows the highest number of direct comparisons. Figure 4 illustrates the results of the NMA for all outcomes separately as well as for the single model using all studies with a prioritization of outcomes.

### Fat mass

Absolute BF mass was the most common outcome, and this was included in 47 studies (74 pairwise comparisons, 12 treatment groups), whereas relative BF mass was included in 37 studies (50 pairwise comparisons, 11 treatment groups). In general, energy restriction combined with any kind of exercise and combined with high-protein content were effective strategies for losing fat mass. The most effective intervention for losing BF in % was energy restriction combined with a high-protein content and exercise (*P* < 0.001, MD: −3.99, 95%: CI −5.44, −2.54). For losing BF in kg, the most effective intervention was energy restriction combined with a high-protein intake (*P* < 0.001, MD: −5.86, 95% CI: −7.34, −4.37). Energy restriction alone also led to significant loss of fat mass, but to a lesser extent (BF in kg: *P* < 0.001, MD: −4.26, 95% CI: −5.35, −3.17, BF in %: *P* < 0.001, MD: −2.69, 95% CI: −3.83, −1.55). Resistance training or mixed exercise alone also enabled the significant loss of BF, but to a considerably lesser extent than energy restriction alone. No significant effect in terms of reducing BF was observed for the 5:2 diet and resistance training combined with a high-protein diet. Aerobic training was only significant in terms of losing BF in % (*P* = 0.02, MD: −1.41, 95% CI: −2.58, −0.25).

### Muscle mass (LBM/FFM)

LBM or FFM was determined as an outcome in 48 studies (72 pairwise comparisons, 12 treatment groups). Only mixed exercise significantly increased LBM or FFM (*P* = 0.0089, SMD: 0.44, 95% CI: 0.11, 0.77). All other interventions managed to preserve muscle mass. However, we observed a nonsignificant trend that energy restriction alone seemed to result in a loss of muscle mass (*P* = 0.189, SMD: −0.23, 95% CI: −0.57, 0.11).

### BMI

For the outcome BMI, 38 studies were available (60 pairwise comparisons, 12 treatment groups). The most effective intervention for BMI reduction was energy restriction combined with resistance training (*P* < 0.001, MD: −2.04, 95% CI: −2.60, −1.49), followed by energy restriction with aerobic training (*P* < 0.001, MD: −1.91, 95% CI: −2.57, −1.25), energy restriction with high-protein content (*P* < 0.001, MD: −1.86, 95% CI: −2.64, −1.07), and energy restriction alone (*P* < 0.001, MD: −1.67, 95% CI: −2.10, −1.23). We found no significant effects on BMI for the interventions of resistance training, resistance training with a high-protein diet, or aerobic training.

### WC

WC was measured in 25 studies (40 pairwise comparisons, 12 treatment groups). All interventions except aerobic training and resistance training with a high-protein diet significantly decreased WC. Highly effective treatments for reducing WC were either energy restriction alone or combined with any kind of exercise or high-protein diet. The most effective treatments were the 5:2 diet (*P* = 0.03, MD: −5.81, 95% CI: −11.17, −0.45) and energy restiction with aerobic training (*P* = 0.001, MD: −4.85, 95% CI: −7.78, −1.93), energy restriction combined with resistance training (*P* < 0.001, MD: −4.58, 95% CI: −6.91, −2.26), energy restriction with high-protein content (*P* < 0.001, MD: −4.46, 95% CI: −7.02, −1.90), energy restriction with mixed exercise (*P* = 0.003, MD: −4.37, 95% CI: −7.20, −1.53), or energy restriction alone (*P* < 0.001, MD: −4.21, 95% CI: −6.15; −2.27).

### All outcomes combined

In the model combining all outcomes according to our prioritization, 65 studies were available (98 pairwise comparisons). The most effective interventions were energy restriction with resistance training (*P* < 0.001, SMD: −1.65, 95% CI: −2.06, −1.24), energy restriction with mixed exercise (*P* < 0.001, SMD: −1.58, 95% CI: −2.06, −1.10), and energy restriction with high-protein content and exercise (*P* < 0.001, SMD: −1.36, 95% CI: −1.87, −0.85). However, BF mass was considered in 58 pairwise comparisons, LBM/FFM only in 3 pairwise comparisons, and WC in 5 pairwise comparisons in this model, meaning that the model mainly emphasized fat mass reduction.

### Inconsistency

We observed no signs of inconsistency in the networks when comparing changes in BF in kg (*P* = 0.859), BF in % (*P* = 0.986), LBM/FFM (*P* = 0.232), and prioritization (*P* = 0.461). However, we observed inconsistencies in the network when comparing changes in BMI (*P* < 0.001) and WC (*P* < 0.001). In the network comparing the change in BMI, it was necessary to remove 3 studies to obtain a *P* value above the level of significance [66,95,106]. In the network comparing the change in WC, we found 3 studies [52,66,101] that contributed to inconsistency; when removing all 3 of them, the network no longer showed signs of inconsistency (*P* = 0.219), but the same overall results were obtained as for the main analysis.

### Subgroup and sensitivity analyses

No considerable differences were observed when only analyzing studies with women or men. An intervention duration of more than 14 wk was associated with more pronounced weight loss, but the order of effectiveness did not change between categories of interventions. Results did not change substantially when excluding studies with a high risk of bias. The subgroup analysis results supported the hypothesis that interventions combining nutrition and exercise most effectively improve body composition and anthropometric parameters.

### Summary of studies not included in the network meta-analysis

In our literature review, we identified 26 studies [[107], [108], [109], [110], [111], [112], [113], [114], [115], [116], [117], [118], [119], [120], [121], [122], [123], [124], [125], [126], [127], [128], [129], [130], [131], [132]] that could not be included in the network meta-analysis. Eleven of these studies were with mixed nutrition and exercise interventions, 3 were with only exercise interventions, and 12 were with only nutritional interventions. All 26 studies were highly heterogeneous in terms of the interventions used; therefore, they are difficult to compare. A detailed overview about the used interventions as well as the main results of the single studies can be found in Supplementary Table 2.

## Discussion

The aim of conducting this systematic review and NMA was to evaluate which nutrition and exercise interventions most effectively improve body composition (fat mass and muscle mass) and anthropometric measures (BMI and WC) in persons with overweight or obesity near or around retirement age. In the NMA models, we identified several effective nutrition and exercise interventions for this target group. A reduction in BF could be best achieved by applying the measures of energy restriction combined with any kind of exercise or with high-protein intake. Energy restriction alone also reduced BF, but to a lesser extent, and on the contrary, energy restriction alone tended to decrease muscle mass. Muscle mass could only be significantly increased with mixed exercise (resistance and aerobic) interventions, but all other interventions that included exercise effectively preserved muscle mass. A decrease in BMI and/or WC could be achieved with nearly every intervention except aerobic training alone, resistance training alone, or resistance training combined with a high-protein diet. Overall, the most effective strategy for loss of fat mass while maintaining or increasing muscle mass was the combination of energy restriction and exercise (resistance or mixed) and/or a high-protein diet.

Energy restriction to achieve a negative energy balance is still a key therapeutic weight loss strategy recommended in evidence-based guidelines [22,133]. However, according to our results, energy restriction alone does not seem to be an appropriate approach for persons in retirement age. Although it results in weight and fat loss, it tends to result in a loss of muscle mass, which may be considered as an adverse effect as it increases risk of disability, metabolic impairments, mortality, or a low quality of life in this group [134]. In addition, LBM loss is a major factor in weight regain, as LBM is the main driver of energy expenditure [135].

Because aging is associated with muscle loss, great efforts should be made to preserve or even increase muscle mass as well as muscle function and quality in aging or aged persons. Our results show that muscle preservation can be effectively achieved by combining resistance training, mixed exercise, or mixed exercise with high-protein content foods with an energy-restricted diet. This result is similar to those of other systematic reviews that concluded that resistance training added to energy-restricted diets prevents muscle loss in other target groups, e.g., in generally older individuals [25,136]. According to our results, resistance training or the combination of resistance and aerobic training yielded much better results than aerobic training alone regarding muscle mass preservation. This result also agrees with those of other studies. A recent systematic review in older adults, for example, concluded that only resistance training could effectively improve muscle strength, whereas aerobic training could not [137].

The term energy-restricted diet is referred to in several different approaches, including low-carbohydrate or low-fat diets, low- and very low-calorie diets, diets using formula products, time-restricted eating, and many more [138]. Our results do not allow us to determine which approach is best because it was not possible to examine all different approaches to reach energy restriction due to the limited number of similar studies that included people near retirement age. However, the specific approach might not be important as long as the energy intake is below the energy requirements (currently 500 to 1000 kcal). For example, a systematic review comparing intermittent and continous energy restrictions in adults did not find different effects, but both forms of energy restriction resulted in similar amounts of weight loss [139]. Whether weight loss is achieved with a moderate, low- or very low-calorie diet also seems to be of secondary importance, but guidelines address concerns that a low- or very low-calorie diet may be less likely to be nutritionally complete [133], also because very low-calorie intake may induce more intense LBM loss [140]. Another systematic review found that low-fat diets are not more successful than higher-fat, low-carbohydrate diets with regard to long-term weight change in adults [141]. The protein amount, source, and quality are also important components of an energy-restricted diet for maintaining muscle [142]. The amino acid composition (and notably the essential amino acid content) and the timing of protein intake (and especially regarding exercise training) [143] but also of other nutrients should be considered, such as vitamin D [144], as well as the fat quality, either alone or combined with other interventions [145].

This evidence implies that several potentially effective ways to restrict energy intake exist. It is likely to be much more important that persons with overweight or obesity find an approach that suits their lifestyle [133]. The time near retirement is a period that offers a unique opportunity for changing lifestyle habits; this time should be used to change diet and behavior in ways that are comfortable over longer periods of time and ensure a healthy diet over the long term to maintain weight loss. Women and men may also react differently to nutritional and exercise interventions [146], but this was not supported by our NMA results because the subgroup analyses separating men and women did not reveal different results from those of the full analysis.

Risk of bias assessment results indicate that risk of bias of the included studies was mostly unclear, i.e., the reporting was very poor in most of the included studies, and risk of reporting bias was mostly unclear or high. Furthermore, only 48% of all identified studies investigating nutritional interventions could be included in the NMA. This was due to the heterogeneity of the nutritional intervention studies (i.e., highly heterogeneous interventions) and the (frequent) lack of a control group with no intervention or usual care. These studies compared similar interventions but yielded few results in terms of observed differences between the study arms. The quality of the studies including the exercise interventions was much higher. Of these, 88% of the exercise studies and 83% of the studies including nutritional interventions combined with exercise interventions could be included in the NMA, demonstrating a much higher homogeneity. This may also be due to the fact that there are many more different food and diet options available to improve body composition than exercise options.

This systematic review and meta-analysis included a considerably high number of studies with the predefined target group and was conducted systematically based on the recommendations in the Cochrane Collaboration Handbook. This NMA allowed an indirect comparison to be included in the statistical model, enhancing the significance and coverage of the whole model [[147], [148], [149]]. However, this study also had some limitations. The method used to group treatments actually treated different treatments as the same, which might have biased our results.

The included studies of this review are heterogeneous, varying in terms of the study duration, the participants’ sex, age and weight, or the country in which the studies were conducted. We found that some of our networks showed evidence of inconsistency. We were able to partly explain the cause of this inconsistency. Although we could not reject the null hypothesis of no inconsistency, this does not imply that the network is consistent. Nevertheless, this study contributes important evidence that should be considered when developing recommendations for improving body composition aimed at persons with overweight and obesity near retirement age.

## Conclusion

The overall results of this NMA indicate that the most effective strategy to improve body composition, i.e., losing fat without increasing risk of sarcopenia in persons with obesity around retirement age, was combining energy restriction with resistance training or with mixed exercise (resistance combined with aerobic exercise) and/or high-protein intake. Without training, an energy-restricted diet with or without added protein helped individuals lose fat mass but also tended to result in losses of muscle mass. To lose fat while preserving muscle, interventions involving aerobic training, intermittent fasting, resistance training combined with a high-protein diet, and energy restriction alone or in combination with a high-protein diet were not suitable, because they either tend to decrease muscle mass or do not reduce BF.

The important life period near retirement provides individuals with an opportunity to start establishing new healthy nutrition and exercise habits and to incorporate evidence-based nutrition and exercise interventions into their daily routines. The main aim is to prevent dependency and disability in older age. Healthcare professionals involved in the management of persons with obesity must be aware that an energy-restricted diet alone probably contributes to the development of sarcopenic obesity in persons of retirement age. To simultaneously lose weight and maintain muscle mass, the combination of energy restriction and resistance training is probably the best way forward.

## Acknowledgments

The authors’ responsibilities were as follows – DE, SB: designed research; MT, SB, DE, LR: conducted research; SE: performed statsistical analysis; DE, SB, LR, MT: wrote the manuscript; JDS, PJW, TV, YB, ACJ: reviewed and edited the manuscript; DE: had primary responibility for the final content; and all authors: read and approved the final manuscript.

### Funding

The SO-NUTS project is funded by JPI HDHL, the funding agencies supporting this work are: the Netherlands Organisation for Health Research and Development (ZonMw), French National Research Agency (ANR), Federal Ministry of Education, Science and Research represented by the Austrian Research Promotion Agency (BMBWF represented by FFG), Spanish State Research Agency (AEI: PCI2020-120683-2) and the Ministry of Education, Youth and Sports Department of Research and Development (MSMT). This project has received funding from the European Union’s Horizon 2020 research and innovation program under the ERA-NET Cofund action No. 727565.

### Author disclosures

The authors report no conflicts of interest.

## Appendix A Supplementary data

Multimedia component1 Multimedia component1
