# Supplementary material for: Nutrition and Exercise Interventions to Improve Body Composition for Persons with Overweight or Obesity Near Retirement Age: A Systematic Review and Network Meta-Analysis of Randomized Controlled Trials
Source: Adv Nutr. 2023 Apr 6;14(3):516–38. doi: 10.1016/j.advnut.2023.04.001 (PMC10201832; doi:10.1016/j.advnut.2023.04.001)
Supplement: Multimedia component1 [file mmc1.docx]

**Nutrition and exercise interventions to improve body composition for persons with overweight or obesity near retirement age: A systematic review and network meta-analysis of randomised controlled trials**

**1^st^ Author: Doris Eglseer**

Supplementary Table 1. Search strings of used databases

| Database | Search string | Results | Date of search^4^ |
| --- | --- | --- | --- |
| PubMed | (("Obesity"[Title/Abstract] OR "Obesity"[MeSH Terms]) AND ("train*"[Title/Abstract] OR "physical activity"[Title/Abstract] OR ("Exercise"[Title/Abstract] OR "Exercise"[MeSH Terms]) OR ("Diet"[Title/Abstract] OR "Diet"[MeSH Terms]))) AND ((randomizedcontrolledtrial[Filter]) AND (middleagedaged[Filter])) | 4.604 | 14th September 2021 |
| EMBASE | ((obesity/^3^) OR (obesity.m_titl.)) AND ("train*".m_titl.) OR ("physical activity".m_titl^2^.) OR (physical activity/) OR (exercise.m_titl.) OR (exercise/) OR (diet.m_titl). OR (diet/))  limit to (adult <18 to 64 years> or aged <65+ years>) | 4.610 | 14th September 2021 |
| CINAHL | (AB^1^ obesity) OR (MH "Obesity")) AND ((AB train*) OR (AB "physical activity) OR (MH "Physical Activity") OR (AB exercise) OR (MH "Exercise") OR (AB diet) OR (MH "Diet"))  Limited publication type randomized controlled Trail, age groups middle aged 45/64 years, aged 65] years | 1.184 | 14th September 2021 |
| CENTRAL | ((obesity.m_titl.) OR (Obesity/)) AND (("train*".m_titl.) OR (exercise.m_titl.) OR ("physical activity".m_titl.) OR (Exercise/) OR (diet.m_titl.) OR (Diet/))  limit 13 to randomized controlled trial | 671 | 15th September 2021 |

^1^AB=title and abstract, ^2^ m_titl =title and abstract,

^3^ /= sub headings, MH = sub headings

^4^ The search was updated on July 12th 2022.

*Supplementary Table 2. Summary of used interventions and results of studies not included in the network meta-analysis (n=26)*

| **Author (Year)** | **Sample Size** | **Intervention** | **Results** |
| --- | --- | --- | --- |
| **Studies which combined nutritional and exercise interventions** | | | |
| Brennan et al. 2020 | 61 | 1. No Intervention (education, normal diet) 2. Energy restriction (500-1000 kcal) 3. Energy restriction + exercise (4-5x/wk AT+ RT) | Energy restriction combined with exercise was more effective to loose weight and body fat than energy restriction alone. |
| Felix-Soriano et al. 2021 | 85 | 1. Olive oil (30 ml or capsule form) 2. Omega-3 fatty acids 3. Omega-3 fatty acids RT (2x/wk) 4. Olive oil + RT (2x/wk) | All interventions improved body weight and fat mass. Only intervention groups with resistance training improved muscle mass. |
| Grossmann et al. 2018 | 11 | 1. Energy restriction + HIIT (3x/wk) 2. Energy restriction + AT (3x/wk) | Energy restriction combined with HIIT led to significant more improvements in weight loss and body composition than aerobic training. |
| Hays et al. 2004 | 34 | 1. No intervention (education, normal diet) 2. Low-fat, complex-carbohydrate (HI-CHO) 3. Low fat, high comlex carbs + exercise (4x/wk HI-CHO + EX) | Both interventions led to significant reduction in relative body fat and body weight than the control group. |
| Jo et al. 2019 | 11 | 1. VLCD (very low-calorie diet) + Optifast + high protein 2. VLCD + RT (3x/wk) + Optifast | Both groups led to a significant reduction of total body mass and fat mass. No significant differences between the groups. |
| Kelly et al. 2014 | 24 | 1. Low glycemic index diet (LoGIX) + exercise (5x/wk) 2. High glycemic index diet (HiGIX) + exercise (5x/wk) | No significant difference in weight loss between the intervention groups. |
| Mulya et al. 2017 | 20 | 1. High glycemic index diet + exercise (HiGIX) 2. Low glycemic index diet (LoGIX) + exercise (5x/wk) | Both interventions did not show significant reduction in BMI, fat mass and body weight. No significant difference in body composition between the intervention groups. |
| Muollo et al. 2019 | 38 | 1. Supervised Nordic walking (3x/wk) + energy restriction 2. Supervised traditional walking (> 3x/wk) + energy restriction | Supervised nordic walking improved BMI and WC and body fat. Not clear, if there were significant differences between the groups. |
| Muollo et al. 2021 | 27 | 1. Unsupervised Nordic walking (3x/wk) + energy restriction 2. Unsupervised traditional walking (> 3x/wk) + energy restriction | Unsupervised walking led to significant reduction in body fat. Not clear, if there were significant differences between the groups. |
| Solomon et al. 2013 | 20 | 1. Aerobic training (> 3x/wk, walking, cycling, etc.) + low-glycemic diet 2. Aerobic training (> 3x/wk, walking, cycling, etc.) + high-glycemic diet | Both interventions led to significant reduction in BMI, relative body fat and body weight than control group. |
| Van Gemert et al. 2015 | 243 | 1. No intervention (education, normal diet) 2. Energy restriction (500-1000 kcal) 3. Exercise (4 h/wk AT + RT) | Loss of body fat was significantly higher in the exercise group compared to the energy restriction group. Interventions had similar effects on weight loss, BMI and WC. |
| **Studies with exercise interventions** | | | |
| Boukabous et al. 2019 | 18 | 1. High intensity training (75 min/wk HIIT) 2. Moderate intensity training (150 min/wk MICT) | No significant differences between high intensity training and moderate intensity training regarding body composition and anthopometric outcomes. |
| Carneiro et al. 2021 | 40 | 1. Low-intensity RT (3x/wk) 2. High-intensity resistance training (3x/wk) | Both interventions led to a significant reduction in body fat. No significant differences in body composition were found between the groups. |
| Izzicupo et al. 2017 | 30 | 1. Traditional walking (> 3x/wk) 2. Nordic walking | Both groups showed a significant decrease in weight. No significant differences were found in between the groups. |
| **Studies with nutritional interventions** | | | |
| Barbour et al. 2015 | 63 | 1. High oleic peanut consumption (male: 84 g/female: 56 g/d) 2. No intervention (education, normal diet) | High oleic peanut consumption slightly increased weight, but there were no differences in body composition between the groups. |
| Barnard et al. 2005 | 59 | 1. Low-fat vegan diet 2. No intervention (education, normal diet) | The low-fat vegan diet led to significantly increased weight loss and reductions in BMI and waist circumference, compared to the control group. |
| Barnard et al. 2022 | 62 | 1. Mediterranean diet 2. Low-fat vegan diet | The low-fat vegan diet led to significantly increased weight loss and reduced fat mass, compared to the Mediterranean diet group. |
| Christensen et al. 2011 | 192 | 1. Very low-calorie diet (VLCD) 2. Low-energy diet (LED) | Both groups led to significant reductions in weight, with no differences between the groups. The very low-calorie diet led to significantly higher reduction in lean tissue and reported more frequently side effects, compared to the control group. |
| Dennis et al. 2010 | 48 | 1. Hypocaloric diet + 500 ml water prior to each daily meal 2. Energy restriction (500-1000 kcal) | Energy restriction together with 500 ml water prior to each daily meal led to significantly greater weight loss, compared to the nonwater group. |
| Goss et al. 2020 | 34 | 1. Very low calorie and carbohydrate diet (< 10%) 2. Low fat diet | The very low calorie and carbohydrate diet had significantly greater weight loss and body fat loss, compared to the low fat diet. |
| Illich et al. 2019 | 135 | 1. No intervention (education, normal diet) 2. Energy restriction + calcium + vitamin D supplement 3. Energy restriction (500-1000 kcal) + low-fat dairy diet | The group using dairy foods showed significantly higher rates of body fat loss and better preservation rates of lean tissue, compared to the control group. |
| Katz et al. 2012 | 46 | 1. Walnuts 56 g 2. No intervention (education, normal diet) | Anthopometrics did not change after walnut consumption. The control group showed significant decrease in BMI and body weight, compared to intervention group. |
| Kristensen et al. 2012 | 72 | 1. Energy restriction + refined wheat 2. Energy restriction + whole-grain wheat | The whole-grain wheat diet led to a significantly greater reduction in body fat, compared to the refined wheat group. No significant differences between groups were found for body weight change. |
| Njike et al. 2015 | 32 | 1. Typical conventional snack food (200 kcal) 2. Nut-based snack bars (NBSB 200 kcal) | The nut-based snack bars led to a significant decrease in body fat, compared to conventional snack food. No differences were observed between the groups in terms of BMI and WC. |
| Shapses et al. 2004 | 58 | 1. Energy restriction + calcium 1,000 mg/d 2. Energy restriction (500-1000 kcal) 3. Energy restriction + calcium 1,000 mg/d (SLIM fast) 4. Energy restriction + Slim Fast | Calcium supplementation did not show a significant effect on weight and body fat for any of the groups. |
| Wien et al. 2003 | 65 | 1. Formula-based low-calorie diet (LCD)+ almond 84 g/d 2. Formula-based low-calorie diet (LCD) + self-selected diet with complex carbs + safflower oil | The almond enriched diet led to a significant higher reduction in weight/BMI, WC and fat mass, compared to the self-selected diet. |

Abreviations: BMI = body mass index, WC = waist circumference


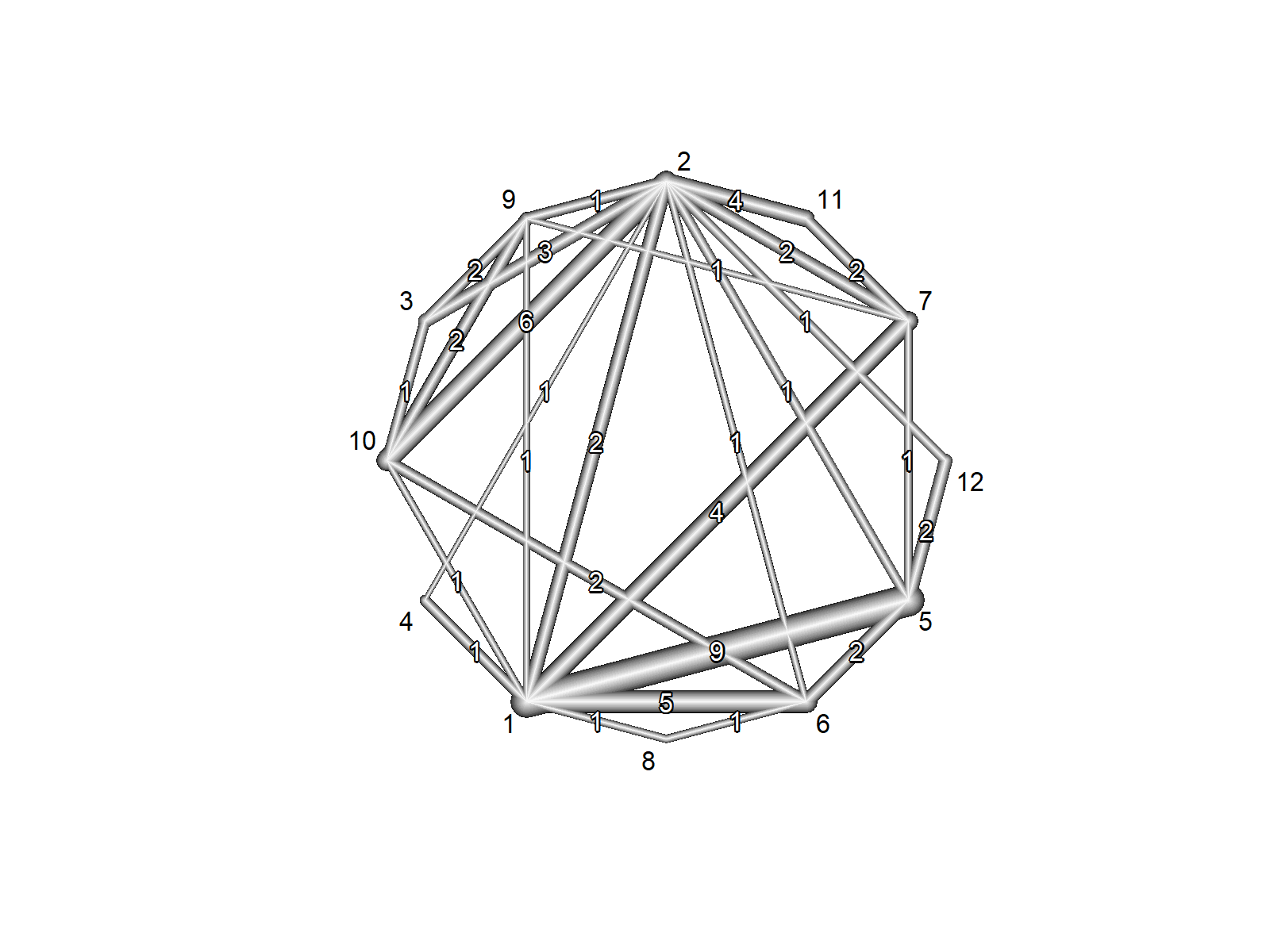

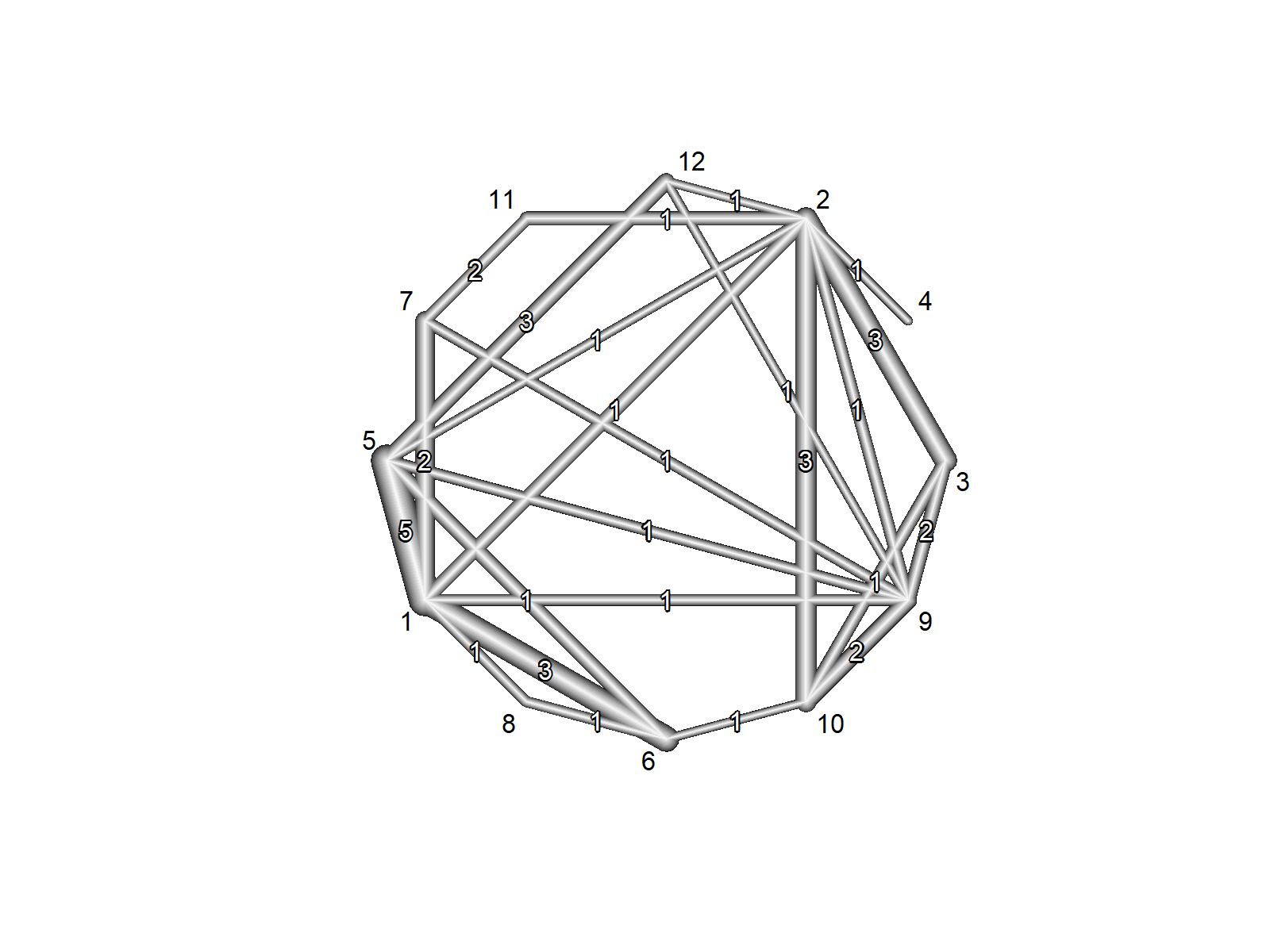

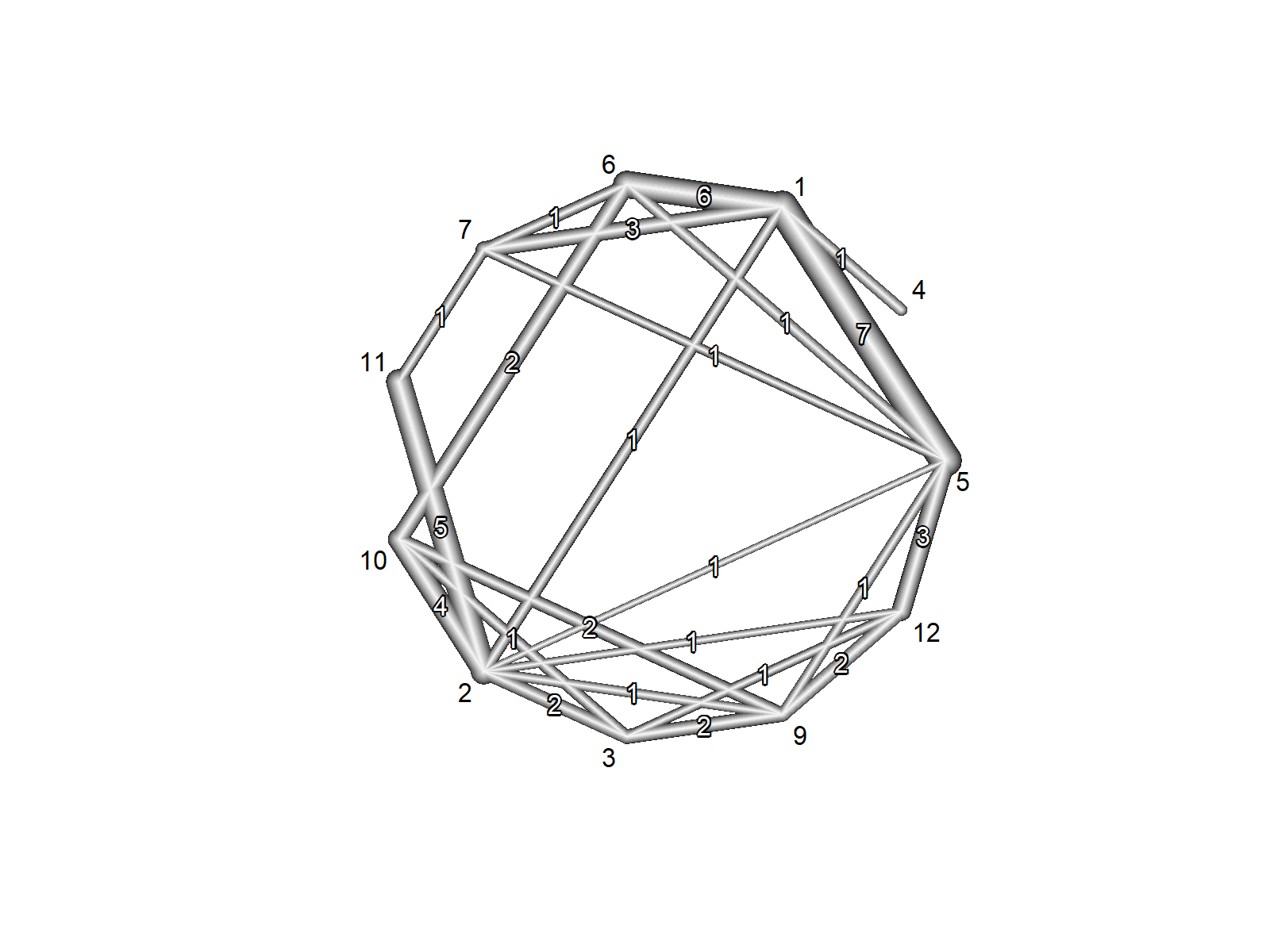


**A** **B** **C**

Supplementary Figure 1. Network graphs comparing the structure of the networks regarding (A) BF%, (B) WC, (C) BMI.

The numbers within the graphs represent the numbers of direct comparisons, while the thickness of the lines is proportional to the inverse standard error of the estimates. The numbers outside the graphs represent the intervention numbers as follows: (1) no intervention, (2) energy restriction, (3) energy restriction plus high protein intake, (4) 5:2 diet, (5) mixed exercise (aerobic and resistance training), (6) resistance training, (7) aerobic training, (8) resistance training plus high protein intake, (9) energy restriction plus high protein and exercise, (10) energy restriction plus resistance training, (11) energy restriction plus aerobic trainig and (12) energy restriction plus mixed exercises.
